# Supplementary material for: EWS-FLI-1 creates a cell surface microenvironment conducive to IGF signaling by inducing pappalysin-1
Source: Genes Cancer. 2017 Nov;8(11-12):762–70. doi: 10.18632/genesandcancer.159 (PMC5755722; doi:10.18632/genesandcancer.159)
Supplement: Supplementary file 1 [file ganc-08-762-s001.pdf]

EWS-FLI-1 creates a cell surface microenvironment conducive to IGF signaling by inducing pappalysin-1 – Jayabal et al

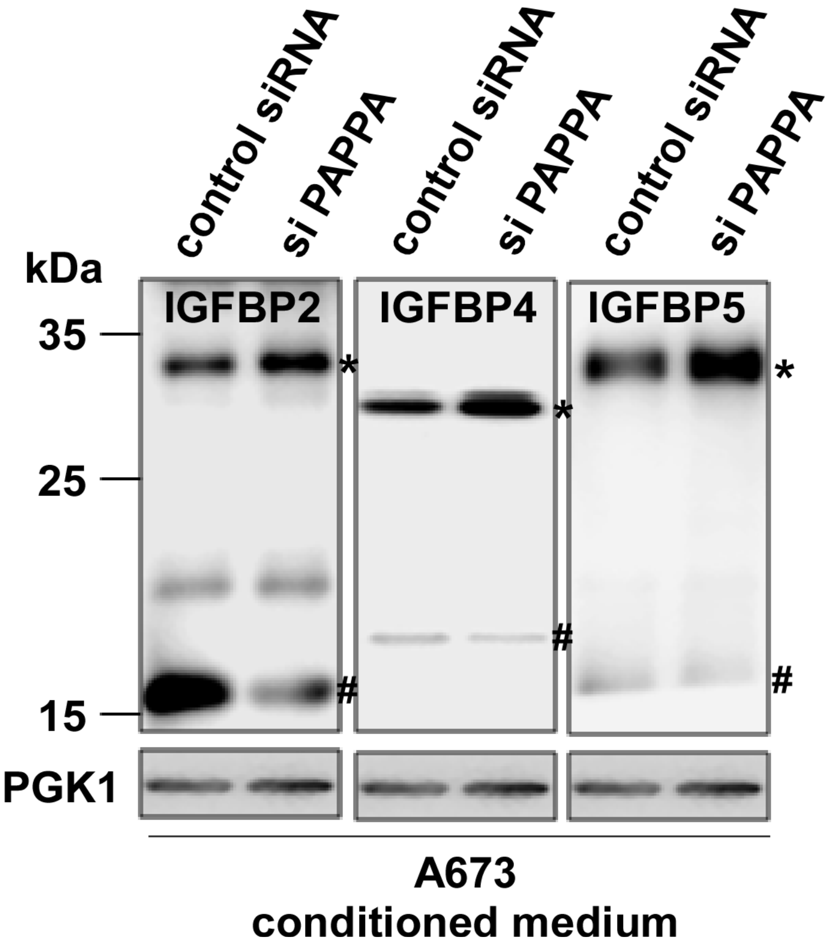

Figure S1: Pappalysin-1 silencing reduces the cleavage products of IGFBP2, IGFBP4, and IGFBP5.

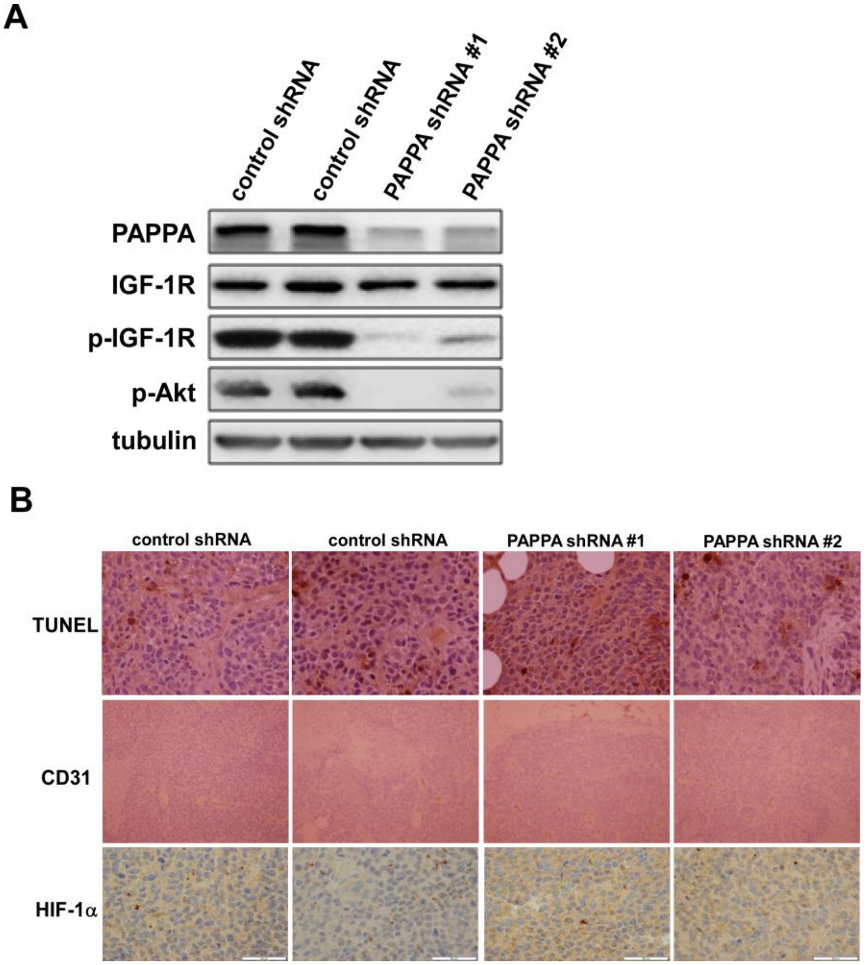

Figure S2: Immunoblotting and immunohistochemistry analysis of xenograft tumors.

(A) Xenograft tumors derived from pappalysin-1 silenced or control shRNA-expressing A673 cells were analyzed for the expression of pappalysin-1, total IGF-1R, phosphorylated IGF-R1, phosphorylated Akt, and tubulin by immunoblotting.

B) The tumor cell apoptosis, tumor angiogenesis, and tumor hypoxia were analyzed by TUNEL assays, CD31 and HIF-1α immunohistochemistry, respectively. Pappalysin-1 silencing had no effect on tumor cell apoptosis, tumor angiogenesis, and tumor hypoxia.
